# Metabolic dysregulation and gut dysbiosis linked to hyperandrogenism in female mice

**DOI:** 10.1002/edm2.443

**Published:** 2023-10-23

**Authors:** Annie Chen, Alex Handzel, Lillian Sau, Laura Cui, Scott T. Kelley, Varykina G. Thackray

**Affiliations:** ^1^ Department of Obstetrics, Gynecology and Reproductive Sciences University of California San Diego, La Jolla California USA; ^2^ Bioinformatics and Medical Informatics Program San Diego State University San Diego California USA; ^3^ Department of Biology San Diego State University San Diego California USA

**Keywords:** androgen, gut microbiome, insulin resistance

## Abstract

**Introduction:**

Polycystic ovary syndrome (PCOS) is a common endocrine pathology in women. In addition to infertility, women with PCOS have metabolic dysregulation which predisposes them to Type 2 diabetes, cardiovascular disease and non‐alcoholic fatty liver disease. Moreover, women with PCOS have changes in their gut microbial community that may be indicative of dysbiosis. While hyperandrogenism is associated with both the development of metabolic dysfunction and gut dysbiosis in females, the mechanisms involved are not well understood.

**Methods:**

We used dihydrotestosterone (DHT) and ovariectomy (OVX) mouse models coupled with metabolic assessments and 16S rRNA gene sequencing to explore the contributions of hyperandrogenism and oestrogen deficiency to the development of insulin resistance and gut microbial dysbiosis in pubertal female mice.

**Results:**

We demonstrated that, while DHT treatment or OVX alone were insufficient to induce insulin resistance during the pubertal‐to‐adult transition, combining OVX with DHT resulted in insulin resistance similar to that observed in letrozole‐treated mice with elevated testosterone and decreased oestrogen levels. In addition, our results showed that OVX and DHT in combination resulted in a distinct shift in the gut microbiome compared to DHT or OVX alone, suggesting that the substantial metabolic dysregulation occurring in the OVX + DHT model was accompanied by unique changes in the abundances of gut bacteria including S24‐7, Rikenellaceae and *Mucispirillum schaedleri*.

**Conclusions:**

While hyperandrogenism plays an important role in the development of metabolic dysregulation in female mice, our results indicate that investigation into additional factors influencing insulin resistance and the gut microbiome during the pubertal‐to‐adult transition could provide additional insight into the pathophysiology of PCOS.

## Graphical abstract

Dihydrotestosterone (DHT) and ovariectomy (OVX) mouse models coupled with metabolic assessments and 16S rRNA gene sequencing were used to explore the contributions of hyperandrogenism alone and in combination with OVX to insulin resistance and gut microbial dysbiosis in pubertal female mice. Our results showed that DHT is insufficient to induce insulin resistance during the pubertal‐to‐adult transition and results in a distinct gut microbial community compared to DHT combined with OVX. These findings emphasize that, while androgens play an key role in PCOS pathology, understanding how additional factors synergize with androgens to induce insulin resistance and differentially regulate the gut microbiome may provide insight into novel therapeutic approaches.

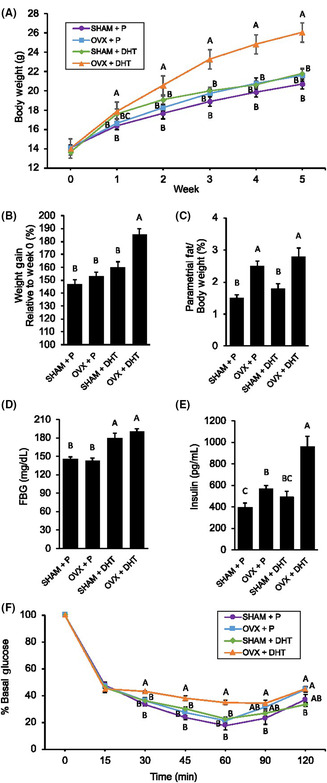

## 1 INTRODUCTION

Polycystic ovary syndrome (PCOS) is a common endocrine disorder affecting approximately 10%–15% of reproductive‐age women.^1^ PCOS diagnosis requires two out of three Rotterdam consensus criteria: hyperandrogenism, oligo−/amenorrhoea and polycystic ovaries.^2^ Reproductive dysfunction is a hallmark of PCOS as patients are at greater risk for infertility and pregnancy complications.^3^, ^4^ Metabolic dysfunction is also associated with PCOS including obesity, insulin resistance, dyslipidaemia and hypertension, all of which increase the risk of developing Type 2 diabetes, cardiovascular disease and non‐alcoholic fatty liver disease.^5^, ^6^, ^7^, ^8^, ^9^, ^10^, ^11^, ^12^ Interestingly, the majority of hyperandrogenic women with PCOS have some degree of insulin resistance that is independent of body mass index,^13^, ^14^ suggesting that the relationship between androgens and insulin resistance may play an important role in PCOS pathophysiology. Additionally, women with PCOS have changes in their gut microbiome, including a decrease in overall diversity and changes in the abundance of specific bacteria.^15^, ^16^, ^17^, ^18^, ^19^, ^20^, ^21^, ^22^ While faecal microbiome transplant studies indicate that the gut microbiome may influence PCOS pathology,^18^ the role of hyperandrogenism in regulating the gut microbial community is poorly understood.

As hyperandrogenism is associated with PCOS, researchers have employed mouse models to study the role of androgen excess in the development and pathology of PCOS.^23^ We previously developed a mouse model that uses letrozole (LET), a nonsteroidal aromatase inhibitor, to limit the conversion of testosterone to oestrogen, resulting in increased testosterone levels and decreased oestrogen levels. This model was based on findings that genetic variants of the aromatase gene were associated with PCOS and that decreased aromatase gene expression and oestrogen/androgen ratio were found in the follicular fluid of women with PCOS.^24^, ^25^, ^26^, ^27^, ^28^ LET treatment recapitulated many reproductive hallmarks of PCOS including hyperandrogenism, acyclicity, polycystic ovaries and elevated LH pulsatility.^29^, ^30^ LET treatment during puberty also resulted in PCOS‐like metabolic dysregulation, including increased weight, abdominal adiposity, increased fasting blood glucose (FBG) levels, hyperinsulinaemia, insulin resistance and dyslipidaemia.^29^, ^31^ In addition, LET treatment resulted in changes in gut microbes and metabolites^32^, ^33^ and exposure to a healthy gut microbiome via a cohousing paradigm improved both reproductive and metabolic phenotypes in the LET model.^34^

Another rodent model used to study the effects of hyperandrogenism is the pubertal dihydrotestosterone (DHT) model.^35^, ^36^ Treatment with exogenous DHT that cannot be aromatized to oestrogen results in reproductive dysfunction, including hyperandrogenism, acyclicity and polycystic ovaries.^37^, ^38^ However, DHT mice lack elevated LH levels that are characteristic of women with PCOS. Although treatment with DHT for 3 months or more was associated with weight gain, increased body fat and glucose intolerance,^37^, ^38^ the short‐term effects of DHT treatment on metabolism or the gut microbiome during the pubertal‐to‐adult transition have not been well characterized.

As testosterone and oestrogen levels are both altered in the LET mouse model and women with PCOS may have altered aromatase activity, we used DHT and ovariectomy (OVX) models to explore the contributions of hyperandrogenism and oestrogen deficiency both independently and in combination to the development of insulin resistance and gut dysbiosis in pubertal female mice. Our results demonstrated that, while DHT treatment alone was insufficient to induce insulin resistance, the combination of DHT with OVX resulted in insulin resistance during the pubertal‐to‐adult transition. In addition, our results showed that DHT and OVX treatment in combination resulted in a distinct shift in the gut microbiome compared to DHT or OVX alone, suggesting that the substantial metabolic dysregulation occurring in the DHT and OVX model is accompanied by unique changes in gut bacteria including S24‐7 and *Mucispirillum schaedleri*. While hyperandrogenism plays an important role in the development of metabolic dysregulation in female mice, our results suggest that investigation into other factors including aromatase activity that could influence insulin resistance and the gut microbiome during the pubertal‐to‐adult transition may provide additional insight into the pathophysiology of PCOS.

## 2 MATERIALS AND METHODS

### 2.1 Mouse models

Three‐week‐old C57BL/6N female mice were purchased from Envigo and housed in a vivarium with an automatic 12 h light:12 h darkness cycle (light period: 6:00 AM–6:00 PM). Mice were given ad libitum access to water and food (Teklad Global 18% Protein Extruded Diet, Envigo) and weighed each week.

At 4 weeks of age, mice underwent either a sham surgery (SHAM) or ovariectomy (OVX). Mice were anaesthetised with 2.5% isoflurane and placed in a prone position on a sterile field. A dorsal incision was made approximately 1 cm from the top of each leg. An incision in the inner layer of skin exposed the ovary near the fat pad which was then removed from the adherent tissue. Absorbable surgical suture (5‐0, 18″ Chromic Gut Absorbable Suture) was used to suture the abdominal cavity/muscle wall. The outer skin incision was closed with surgical wound clips. Mice that were sham‐operated were subject to similar surgical procedures, but the ovary was not removed.

Additionally, at 4 weeks of age, the mice were implanted subcutaneously with either a placebo (P) or DHT (Steraloids) implant made in‐house with an 8 mm silastic implant (i.d., 1 mm; o.d., 2.15 mm, 1118915D, Dow Corning) containing 2 mg of DHT within a 4 mm space. Surgery and implant combinations created four experimental groups: SHAM + P, OVX + P, SHAM + DHT and OVX + DHT (*n* = 10/group).

### 2.2 Oestrous cycle assessment

Oestrous cycles of the mice were determined during Weeks 4–5 of treatment by light microscopy examination of vaginal cytology for 7 days, as previously described.^39^ Dioestrus consisted predominantly of leucocytes; pro‐oestrus of nucleated epithelial cells; oestrus of cornified epithelial cells; metoestrus of a combination of cornified and nucleated epithelial cells along with leucocytes.

### 2.3 Insulin tolerance test

At 5 weeks of treatment, mice were fasted for 5 h. A handheld glucometer (One Touch UltraMini; LifeScan, Inc.) was used to measure FBG at all timepoints. Tail blood was collected and FBG was measured at time point 0 before administration of an intraperitoneal injection of insulin (0.75 U/kg in sterile saline, Humulin R U‐100). Blood glucose was measured at 15, 30, 45, 60, 90 and 120 min post insulin injection. Collected tail blood was used to measure serum fasting insulin levels via ELISA at the UC Davis Mouse Metabolic Phenotyping Center.

### 2.4 Tissue collection

Five weeks post implantation, mice were euthanized with 2.5% isoflurane. Terminal blood was collected from the inferior vena cava for hormone assays. Parametrial fat pads were dissected and weighed.

### 2.5 Hormone assays

Radioimmunoassays were used to measure serum LH levels (range 0.02–75 ng/mL) at the University of Virginia Center for Research in Reproduction Ligand Assay and Analysis Core Facility. Serum DHT levels (range 0.093–750 ng/mL) were measured by radioimmunoassay at the Oregon National Primate Research Center Endocrine Technologies Core.

### 2.6 Statistical analyses of reproductive and metabolic phenotypes

The statistical package JMP 15 was used to analyse differences among groups. Data residuals were checked for normality and one‐way ANOVA followed by post hoc Tukey–Kramer honestly significant difference test was performed. If residuals were not normal, data underwent Box‐Cox transformation. Non‐parametric tests were performed if transformation did not result in normality. Statistical significance (*p* < .05) was indicated by different letters or an asterisk.

### 2.7 Faecal sample collection, DNA isolation and 16S rRNA gene sequencing

Faecal samples were collected from 10 mice/group (40 mice total) at 4 weeks of age prior to treatment and once per week during the 5 weeks of the experiment without regard to the oestrous cycle stage. Faecal samples were frozen and stored at −80°C. DNA was extracted from the faecal samples using the DNeasy PowerSoil Pro kit (Qiagen). Samples were amplified using polymerase chain reaction using 515F and 806R to target the V4 region of 16S rRNA.^40^ Amplicon sequencing libraries were prepared at the Scripps Research Institute Next Generation Sequencing Core Facility and samples were sequenced on an Illumina MiSeq Platform as previously described.^32^

### 2.8 Bioinformatics analysis of 16S rRNA sequences

Processing of the forward sequence reads was performed using Qiime2 (version 2021.4.0). Demultiplexing resulted in 8.7 million sequences with an average of 35,223 sequences per sample. DADA2 was used for denoising the sequence data and to create sequence variants (SVs).^41^ Sequences were truncated to 280 bp (when the average quality score dropped below 20). For taxonomic analysis, the function feature‐classifier classify‐sklearn was trained on Greengenes 13_8 99% and applied to the sequences. For beta diversity, the feature table was exported and converted to a biom‐formatted SV table. SVs were then removed if they were present in four or fewer samples. As microbiome data are compositional by nature,^42^, ^43^ the SV table was center log‐ratio (clr) transformed to convert the data from compositional to a N‐dimensional Euclidean vector subspace. Eight samples that contained >30% of k__bacteria (unknown) were removed from the SV table. This resulted in an SV table of 4426 SVs with 232 samples.

### 2.9 Statistical analysis of 16S rRNA gene sequencing

Statistical analyses were performed in the R statistical package (version 4.2.1). Nonmetric multidimensional scaling (NMDS) was used in the R package Vegan (version 2.6‐2) to visualize differences among the treatment groups over time. To statistically analyse differences among treatment groups, permutational multivariate analysis of variance using distance (PERMANOVA) was used (9999 permutations ‘vegan’ package). The R package Random Forest (version 4.7‐1.1) was used to determine the top 50 features that best predicted the treatment group based on Mean Decrease Gini.^44^ These features were used for differential taxa analysis using a linerar mixed model using the nlme package in R (version 3.1‐158) with treatment and time as the independent variables and the individual mouse as the random effect. The model was Feature ~ Treatment*Time and the Random Effect was approximately 1 | Mouse ID. A Type I ANOVA was used to determine whether treatment, time or an interaction had significant effects on the feature. The emmeans package from R was utilized to compute estimated marginal means (least‐squares means) for the model to compare treatment groups. The *p*‐values for treatment, time and the interaction were adjusted using the FDR method.

## 3 RESULTS

### 3.1 Mice with ovariectomy and DHT treatment have hyperandrogenism and acyclicity

**FIGURE 1:**
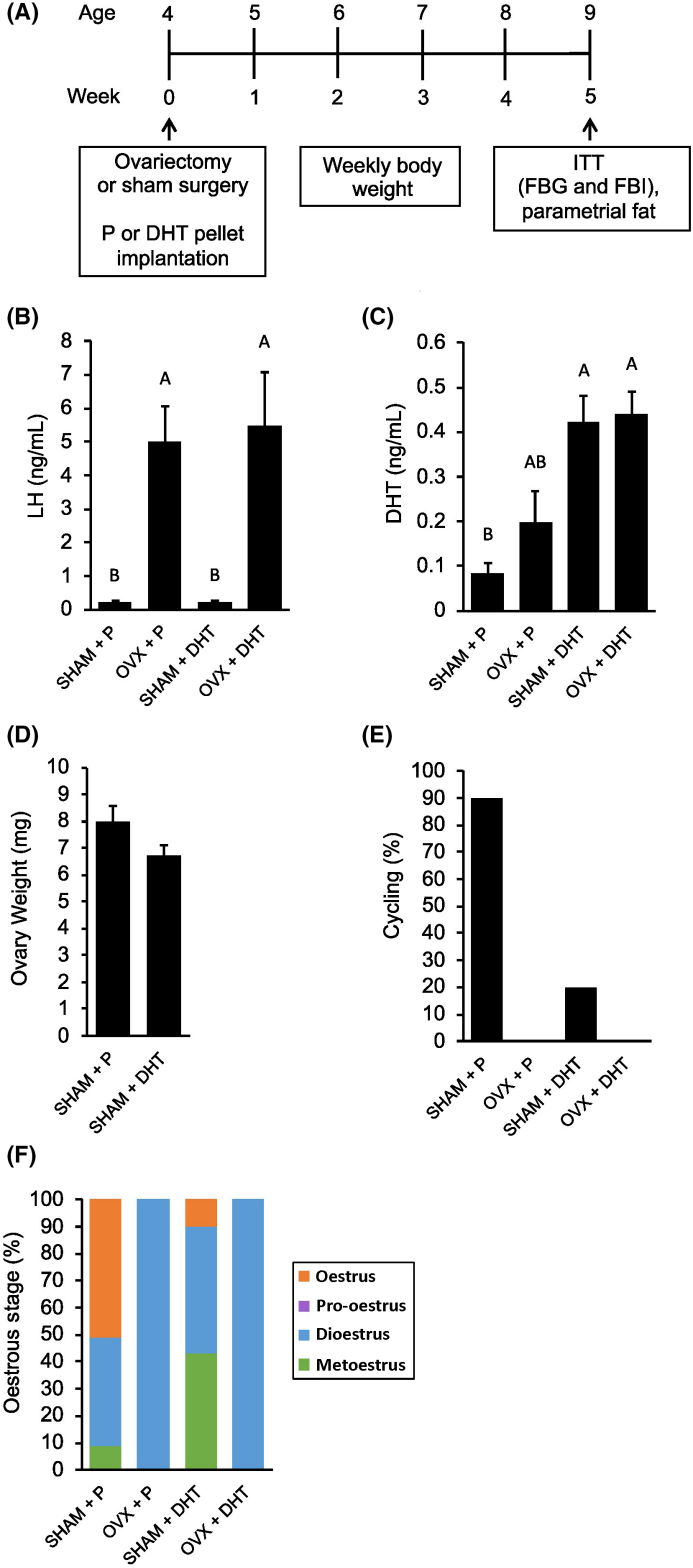
Mice with ovariectomy (OVX) and dihydrotestosterone (DHT) treatment developed acyclicity. (A) Schematic of study design: Mice were given either a placebo (P) or DHT silastic implant, along with either an OVX or sham surgery (SHAM) at 4 weeks of age (*n* = 10 per group). Weekly weight assessments, an insulin tolerance test (ITT), measurement of fasting blood glucose (FBG), fasting blood insulin (FBI) and parametrial fat weight were performed. (B) OVX + P and OVX + DHT mice demonstrated elevated serum luteinising hormone (LH) levels compared to SHAM + P and SHAM + DHT mice, indicating a lack of oestrogen negative feedback. (C) SHAM + DHT and OVX + DHT mice had increased serum DHT levels compared to SHAM + P. (D) There was no difference in ovary weight between SHAM + P and SHAM + DHT mice. (E, F) All OVX + DHT mice were acyclic and were stuck in the dioestrus stage. Graph error bars represent standard error of the mean. Different letters signify significant differences among groups using a one‐way ANOVA followed by post hoc comparisons with the Tukey–Kramer honestly significant difference test (*p* < .05).

As the contribution of hyperandrogenism to metabolic dysregulation in female mice is still unclear, we investigated whether elevated androgens were sufficient to induce insulin resistance and gut dysbiosis alone or in combination with OVX during the pubertal‐to‐adult transition. Four‐week‐old mice were either OVX or SHAM and treated with either P or DHT for 5 weeks (Figure 1A). OVX + DHT mice had elevated levels of serum LH similar to OVX + P mice, indicating lack of ovarian negative feedback (Figure 1B). As expected, repressive effects of DHT on LH pulses in the context of OVX were not observed with a one‐off measurement of LH in contrast to repeated LH sampling.^45^ OVX + DHT mice also had elevated serum levels of DHT similar to SHAM + DHT (Figure 1C). No significant difference was observed in ovary weight between the two SHAM groups treated with P or DHT (Figure 1D). Also, like OVX and DHT mice, mice with both OVX + DHT lacked regular oestrous cycles and they were stuck in the dioestrus stage (Figure 1E,F).

### 3.2 Ovariectomized and DHT‐treated mice displayed a PCOS‐like metabolic phenotype that included insulin resistance

**FIGURE 2:**
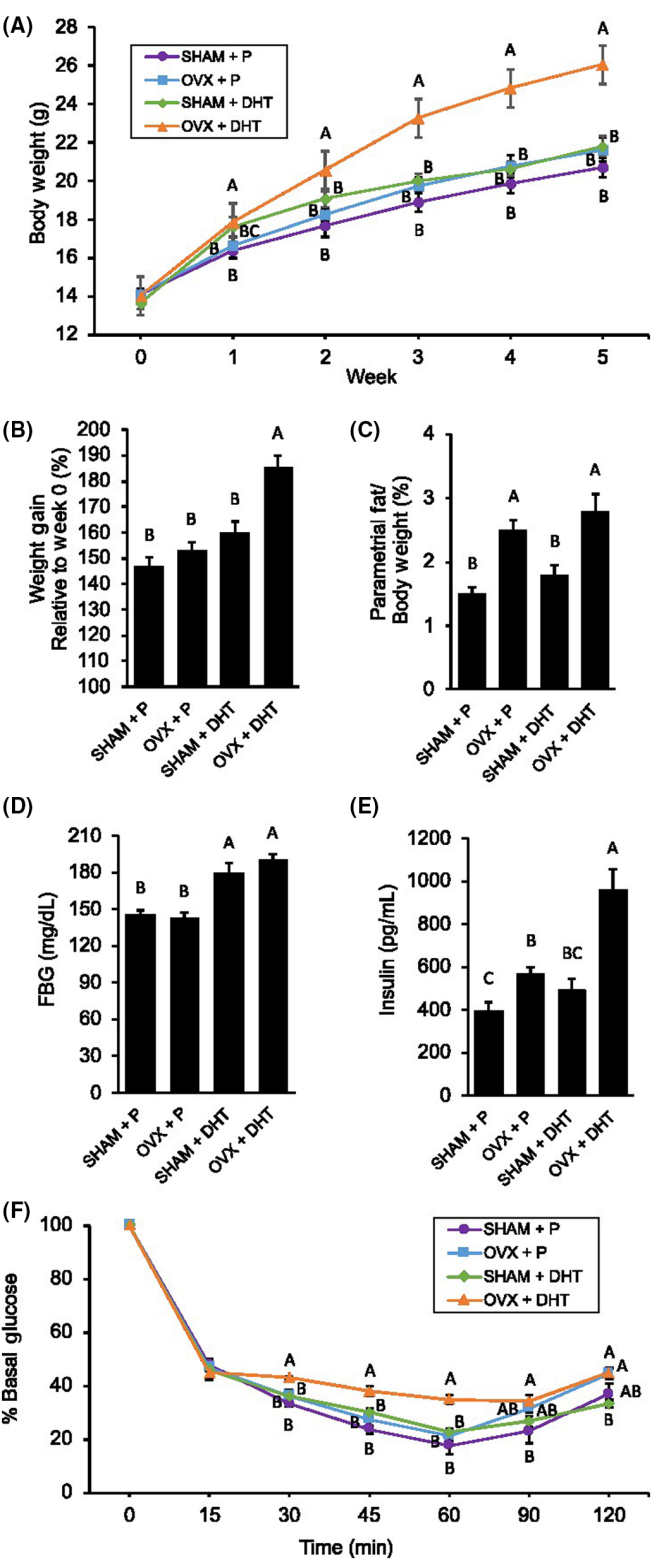
Ovariectomy (OVX) and dihydrotestosterone (DHT) treatment resulted in a more severe metabolic phenotype compared to OVX or DHT treatment alone. (A, B) OVX + DHT mice gained significantly more weight compared to the other three groups. (C) Similar to OVX + placebo (P) mice, OVX + DHT mice had greater parametrial fat relative to body weight compared to sham surgery (SHAM) + P and SHAM + DHT. (D) Similar to SHAM + DHT mice, OVX + DHT mice had significantly higher fasting blood glucose (FBG). (E) OVX + DHT mice had significantly elevated levels of fasting insulin compared to the other three groups. (F) Among the four groups, only OVX + DHT mice displayed insulin intolerance. Graph error bars represent standard error of the mean. Different letters signify significant differences among groups using a one‐way ANOVA followed by post hoc comparisons with the Tukey–Kramer honestly significant difference test (*p* < .05).

One week after surgery, OVX + DHT mice gained significantly more weight than the other three groups, maintaining this trend until the end of the experiment (Figure 2A,B). In addition to weight gain, OVX + DHT mice also had greater abdominal adiposity compared to SHAM + P as reflected by an increase in parametrial fat relative to body weight that was similar to OVX + P mice (Figure 2C). As for glucose homeostasis, OVX + DHT mice had elevated FBG and were severely hyperinsulinemic (Figure 2D,E). Notably, OVX + DHT mice displayed decreased insulin sensitivity during an ITT compared to the other three groups that did not display insulin resistance (Figure 2F).

### 3.3 DHT treatment and OVX alone or in combination resulted in distinct changes in gut bacterial beta diversity compared to control

**FIGURE 3:**
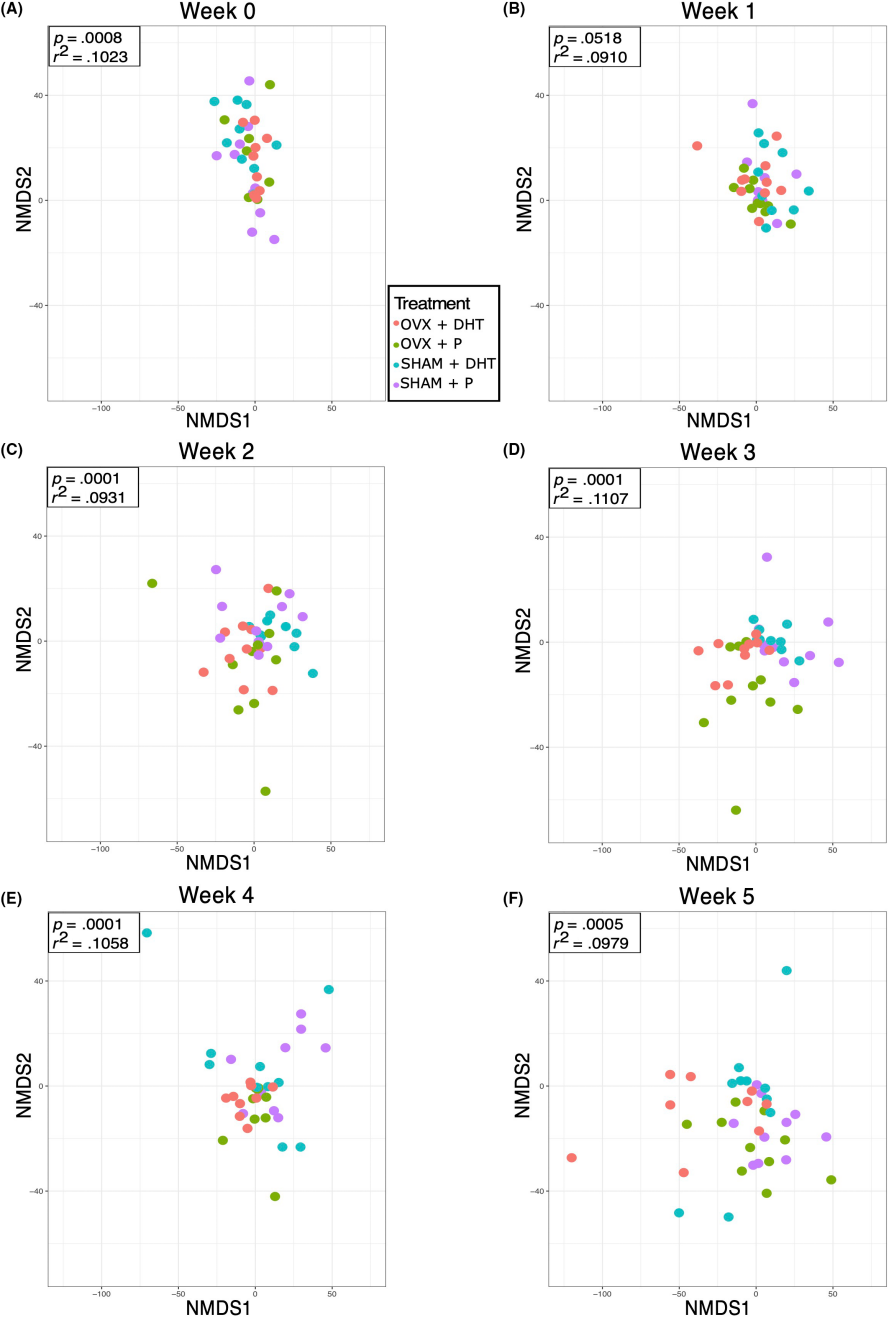
Ovariectomy (OVX) and dihydrotestosterone (DHT) treatment alone or in combination resulted in distinct changes in gut microbial community composition of pubertal female mice 2–5 weeks after treatment. Non‐metric multidimensional scaling (NMDS) plots were used to visualize differences among the four treatment groups: sham surgery (SHAM) + placebo (P), SHAM + DHT, OVX + P and OVX + DHT. The *p*‐value and *r* ^2^ value from a PERMANOVA analysis are shown in the top left corner of each graph.

NMDS was used to visualize differences in beta diversity of gut bacterial communities among the treatment groups at Weeks 0–5 (Figure 3A–F). Analysis of variance (PERMANOVA) determined that the treatment groups differed from each other at Week 0 and Weeks 2–5 (*p* < .05). Analysis of the *R* ^2^ values showed that 9%–11% of the variation in beta diversity was explained by treatment at any given time point. Pairwise PERMANOVA comparisons of beta diversity between treatments were performed for Weeks 1–5 (Table 1). At Week 1, none of the comparisons were different. At Week 2, all pairwise comparisons were different except for the SHAM + P versus OVX + P comparison. At Weeks 3–4, all comparisons were different. At Week 5, all pairwise comparisons were different except for the SHAM + P versus OVX + P and SHAM + DHT versus OVX + DHT comparisons.

**TABLE 1:** Pairwise PERMANOVA comparisons of beta diversity between treatment groups by week.

Treatment	Week 1		Week 2		Week 3		Week 4		Week 5	
	*R* ^2^	*p*‐Value^a^	*R* ^2^	*p*‐Value^a^	*R* ^2^	*p*‐Value^a^	*R* ^2^	*p*‐Value^a^	*R* ^2^	*p*‐Value^a^
SHAM + P vs. OVX + P	.0601	.1400	.0579	.094	.0763	.0001**	.0722	.0004**	.0570	.1143
SHAM + P vs. SHAM + DHT	.0645	.0888	.0644	.0016**	.0687	.0046**	.0607	.0349*	.0640	.0065**
SHAM + P vs. OVX + DHT	.0705	.0861	.0603	.0230*	.0863	.0001**	.0703	.0009**	.0831	.0033**
OVX + P vs. SHAM + DHT	.0572	.2900	.0719	.0004**	.0810	.0001**	.0876	.0001**	.0665	.0025**
OVX + P vs. OVX + DHT	.0611	.2064	.0603	.0288*	.0650	.0018**	.0826	.0001**	.0692	.0284*
SHAM + DHT vs. OVX + DHT	.0619	.3268	.0705	.0005**	.0825	.0004**	.0690	.0023**	.0657	.0601

^a^ Corrected for multiple comparisons within week via the Benjamini–Hochberg method.

*p‐values < .05; **p‐values < .01.

### 3.4 DHT treatment and OVX alone or in combination resulted in distinct changes in gut bacteria including *Lactobacillus*, S24‐7, Rikenellaceae and *Mucispirillum schaedleri*

The Random Forest algorithm was used to identify the top 50 bacterial taxa with the most predictive power for differentiation among the four treatment groups. A linear mixed‐effects model was then used to determine which of these 50 taxa differed by treatment, time or interaction between treatment and time. Treatment was found to alter the abundance of 27 bacteria after adjusting for multiple comparisons including *Lactobacillus*, S24‐7, Rikenellaceae and *Mucispirillum schaedleri* (Table 2). Twenty taxa were from the Bacteroidetes phylum, five were Firmicutes and two were Deferribacteres. Nineteen of these bacteria were affected by time while only four were affected by an interaction between treatment and time. Pairwise comparisons between means determined which treatments had different bacterial relative abundances (Table 3). The OVX + P versus SHAM + DHT comparison had the most different bacteria with a total of 13 out of 27. This was followed by the OVX + DHT versus SHAM + DHT comparison with 12 out of 27.

**TABLE 2:** Statistical analysis using a linear mixed effect model of clr‐transformed bacterial abundances (SVs significantly different by treatment).

Phylum	Classification	Treatment, *p*‐value	Time, *p*‐value^a^	Interaction, *p*‐value^a^
Firmicutes	*Lactobacillus*	.00001*	.01014*	.71993
Firmicutes	*Lactobacillus*	.00001*	.01031*	.22436
Firmicutes	*Lactobacillus*	.00001*	.01606*	.33317
Bacteroidetes	Bacteroidales	.00004*	.00555*	.70922
Bacteroidetes	S24‐7	.00013*	.06724	.21414
Bacteroidetes	Bacteroidales	.00036*	.00670*	.91186
Firmicutes	*Lactobacillus*	.00037*	.00440*	.56983
Bacteroidetes	Bacteroidales	.00087*	.03516*	.65759
Bacteroidetes	Rikenellaceae	.00087*	.08794	.03934*
Bacteroidetes	S24‐7	.00158*	.05580	.15555
Bacteroidetes	Rikenellaceae	.00222*	.01182*	.14959
Firmicutes	*L. vaginalis*	.00255*	.04379*	.04812*
Deferribacteres	*M. schaedleri*	.00603*	.00634*	.25809
Bacteroidetes	S24‐7	.01299*	.00440*	.22436
Bacteroidetes	Rikenellaceae	.02572*	.38709	.16063
Bacteroidetes	S24‐7	.02572*	.00120*	.17192
Bacteroidetes	S24‐7	.02572*	.00850*	.17192
Bacteroidetes	S24‐7	.02884*	.01335*	.03934*
Bacteroidetes	Bacteroidales	.02956*	.00761*	.47706
Deferribacteres	*M. schaedleri*	.03092*	.00428*	.08629
Bacteroidetes	Bacteroidales	.03998*	.09314	.16550
Bacteroidetes	Rikenellaceae	.03998*	.00555*	.20091
Bacteroidetes	S24‐7	.04026*	.00555*	.47706
Bacteroidetes	Rikenellaceae	.04077*	.13394	.03934*
Bacteroidetes	Bacteroidales	.04717*	.06420	.07749
Bacteroidetes	Bacteroidales	.04717*	.01217*	.19256
Bacteroidetes	Bacteroidales	.04799*	.00923*	.25809

Abbreviations: *L. vaginalis*, *Lactobacillus vaginalis*; *M. schaedleri*, *Mucispirillum schaedleri*.

^a^ The linear model also included time (weeks) and treatment by time interaction terms.

* *p*‐Values < .05 and were adjusted for multiple comparisons via the Benjamini–Hochberg method.

**TABLE 3:** Pairwise comparisons between treatments from the linear mixed effects model.

Phylum	Classification	OVX + DHT vs. OVX + P	OVX + DHT vs. SHAM + DHT	OVX + DHT vs. SHAM + P	OVX + P vs. SHAM + DHT	OVX + P vs. SHAM + P	SHAM + DHT vs. SHAM + P
Firmicutes	*Lactobacillus*	0.0312*	0.9290	0.8484	0.0066*	0.0037*	0.9970
Firmicutes	*Lactobacillus*	0.0144*	0.9916	0.9988	0.0068*	0.0094*	0.9991
Firmicutes	*Lactobacillus*	0.0147*	0.9607	0.9740	0.0040*	0.0402*	0.7963
Bacteroidetes	Bacteroidales	0.4089	0.0358*	0.9622	0.0005*	0.6965	0.0104*
Bacteroidetes	S24‐7	0.7075	0.9763	0.0606	0.9094	0.4391	0.1438
Bacteroidetes	Bacteroidales	0.2024	0.0039*	0.9995	0.3581	0.1641	0.0028*
Firmicutes	*Lactobacillus*	0.0256*	0.3638	0.7886	0.0003*	0.0021*	0.8853
Bacteroidetes	Bacteroidales	0.7265	0.1685	0.9900	0.0167*	0.8799	0.0905
Bacteroidetes	Rikenellaceae	0.8407	0.2773	0.9958	0.0548	0.9303	0.1868
Bacteroidetes	S24‐7	0.4207	1.0000	0.0703	0.4361	0.7641	0.0746
Bacteroidetes	Rikenellaceae	0.9916	0.0166*	0.0366*	0.0348*	0.0727	0.987
Firmicutes	*L. vaginalis*	0.0500	0.9900	0.9737	0.0243*	0.1213	0.8836
Deferribacteres	*M. schaedleri*	0.0022*	0.0053*	0.0131*	0.9869	0.9018	0.9848
Bacteroidetes	S24‐7	0.9038	0.0056*	0.0877	0.0009*	0.0183*	0.6722
Bacteroidetes	Rikenellaceae	0.8423	0.8164	0.9697	0.3379	0.9812	0.5531
Bacteroidetes	S24‐7	0.0366*	0.0002*	0.0000*	0.2634	0.0925	0.9462
Bacteroidetes	S24‐7	0.0479*	0.0144*	0.0015*	0.9639	0.5814	0.8510
Bacteroidetes	S24‐7	0.0174*	0.8885	0.0209*	0.0924	0.9996	0.1091
Bacteroidetes	Bacteroidales	0.7241	0.0241*	0.9993	0.0015*	0.6493	0.0317*
Deferribacteres	*M. schaedleri*	0.0004*	0.0013*	0.0023*	0.9721	0.913	0.9962
Bacteroidetes	Bacteroidales	0.7608	0.4291	0.9382	0.0755	0.9765	0.1677
Bacteroidetes	Rikenellaceae	0.0019*	0.0024*	0.0105*	0.9997	0.9163	0.9450
Bacteroidetes	S24‐7	0.8356	0.1877	0.0007*	0.6280	0.0084*	0.1396
Bacteroidetes	Rikenellaceae	0.6838	0.6010	0.9497	0.1035	0.9376	0.2966
Bacteroidetes	Bacteroidales	0.5861	0.4042	0.9991	0.0354*	0.5015	0.4767
Bacteroidetes	Bacteroidales	0.9669	0.0052*	0.9492	0.0183*	0.9999	0.0206*
Bacteroidetes	Bacteroidales	0.7643	0.0270*	0.6838	0.0021*	0.1766	0.2689

Abbreviations: *L. vaginalis*, *Lactobacillus vaginalis*; *M. schaedleri*, *Mucispirillum schaedleri*.

* *p*‐Values < .05.

**FIGURE 4:**
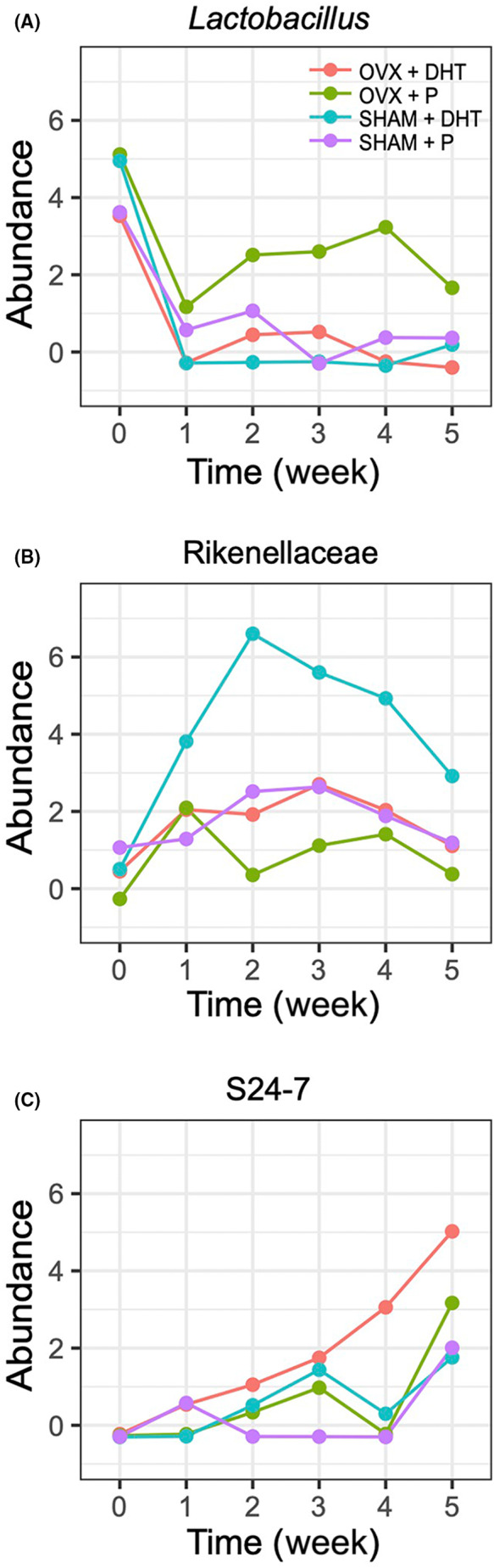
Differential abundance of representative bacteria taxa that changed in the four treatment groups: sham surgery (SHAM) + placebo (P), SHAM + dihydrotestosterone (DHT), ovariectomy (OVX) + P and OVX + DHT. Average clr‐transformed relative abundances (Abundance) were plotted and the SVs were identified at the lowest possible taxonomic level.

Changes in clr‐transformed bacterial abundances over time associated with treatment were graphed for three bacteria of interest including *Lactobacillus*, Rikenellaceae and S24‐7 (Figure 4). The abundance of *Lactobacillus* sp. changed in the OVX + P group while Rikenellaceae showed the largest increase in abundance for the SHAM + DHT group and S24‐7 showed the largest increase in abundance for the OVX + DHT group compared to the other treatment groups.

## 4 DISCUSSION

To investigate the role of hyperandrogenism during puberty in female metabolism and gut dysregulation, we characterized the effects of DHT treatment alone or in combination with OVX during the pubertal‐to‐adult transition. The 4 mm dose was modelled on previous studies that reported a twofold increase in serum DHT in 8‐week‐old adult mice.^46^, ^47^ However, treatment of pubertal female mice with 4 mm DHT resulted in a sevenfold increase in DHT after 5 weeks which is higher than that observed in PCOS and more comparable with 10‐fold higher levels in the previously published mouse model.^37^ DHT treatment resulted in reproductive dysfunction including acyclicity similar to previous studies in pubertal DHT models.^37^, ^48^

In terms of metabolic dysfunction, our results showed that 4 mm DHT treatment for 5 weeks during the pubertal‐to‐adult transition resulted in elevation in FBG and FBI but minimal weight gain and adiposity. These results are in agreement with previous studies showing that DHT causes minimal metabolic effects in the first month of treatment and more dysregulation including insulin resistance with longer treatment (3 months).^37^, ^49^ In contrast to DHT, OVX resulted in increased abdominal adiposity and hyperinsulinemia after 5 weeks but weight gain and dysglycaemia were minimal in this time frame. Notably, insulin resistance did not occur in female mice after 5 weeks of treatment with either 4 mm DHT or OVX, in agreement with a previous study that OVX was not sufficient to induce insulin resistance during the pubertal‐to‐adult transition in female mice.^50^

OVX + DHT combined treatment led to a severe metabolic phenotype that was not observed with OVX or DHT alone. OVX + DHT mice gained weight after just 1 week of treatment, and by Week 5, they were 5–6 g heavier and gained at least 30% more weight compared to the control group. Substantial abdominal adiposity, dysglycaemia and hyperinsulinemia were observed in OVX + DHT mice during the pubertal‐to‐adult transition along with insulin resistance, which was not observed in either DHT or OVX mice but was similar to the phenotype of the LET model.^31^ Our results indicate that hyperandrogenism is not sufficient to induce insulin resistance in certain situations. This idea is supported by the fact that prenatal exposure to androgens did not induce insulin resistance in female offspring as defined by an ITT or hyperinsulinaemic–euglycaemic clamp.^37^, ^51^, ^52^ In addition, LET treatment of adult female mice resulted in a mild metabolic phenotype including a lack of insulin resistance.^53^

In addition to studying the role of hyperandrogenism on metabolism, we used 16S rRNA gene sequencing to study the effects of DHT alone and in combination with OVX on the gut microbiome of pubertal female mice. The beta diversity of gut bacteria differed at Week 0 prior to treatment, perhaps due to slight differences in the gut microbiome of mice shipped from Envigo. These changes resolved by Week 1 of the study so it is likely that they did not influence changes in gut bacteria observed in Weeks 2–5. It is interesting that changes in overall gut bacteria composition were detectable after 2 weeks of DHT, OVX or OVX + DHT treatment, which is similar to LET treatment,^32^, ^33^ indicating that elevated androgens and oestrogen insufficiency due to OVX appear to exert detectable effects on gut microbes in a similar time frame.

A handful of prior studies have demonstrated an effect of elevated androgens or OVX on the gut microbiome in female rodents although the effects on specific bacterial abundance are not consistent. For instance, 6–12 weeks of DHT treatment in pubertal female rats resulted in a shift in beta diversity in addition to an increase in the relative abundance of bacterial taxa including *Clostridium*, *Ruminococcus* and *Alistipes* ^54^ or *Prevotella*.^55^ Furthermore, 3 months of DHT treatment in pubertal female mice led to a shift in beta diversity, an increase in specific Clostridiales, Proteobacteria and Erysipelotrichaceae bacteria, and a decrease in *Bacteroides*, *Parabacteroides* and Porphyromonadaceae.^56^ In addition, a study showed changes in beta diversity, an increase in *Lactobacillus*, and decrease in *Bacteroides acidifaciens*, *Prevotella*, S24‐7 and Rikenellaceae species in adult female mice 12 weeks after OVX^57^ while another study showed changes in beta diversity, an increase in *Allobaculum*, *Ruminococcus* and *Methanobrevibacter* and a decrease in *Prevotella* in adult female rats 16 weeks after OVX.^58^

While it is evident that treatment with androgens or OVX can modulate the gut microbiome, there have been no studies that compared the effects of these treatments on the gut microbiome of female mice during the pubertal‐to‐adult transition. As the metabolic phenotypes of mice with 5 weeks of DHT treatment or OVX overlapped, we expected that the diversity of their gut microbiomes might be more similar when compared to the OVX + DHT microbiome. However, pairwise analysis of beta diversity showed that each treatment group was different from the others for weeks 2–5, except for SHAM + P versus OVX + P at week 2 and SHAM + DHT versus OVX + DHT at Week 5. This indicates that treatment with DHT, OVX or OVX + DHT resulted in distinct gut bacteria communities relative to each other and placebo control mice.

In agreement with the beta diversity analysis, the linear mixed‐effects model highlighted distinct types of bacteria with altered abundances after treatment compared to SHAM + P control. Similar to a previous OVX study in adult mice,^57^ the abundances of multiple *Lactobacillus* and S24‐7 differed between OVX and control. DHT treatment shifted the abundances of members of the Bacteroidales relative to control mice, while the abundances of members of S24‐7 and Rikenellaceae and strains of *M. schaedleri* differed between OVX + DHT and control. Abundances of Bacteroidales, S24‐7, Rikenellaceae and *M. schaedleri* also differed between DHT and OVX + DHT, emphasising the distinct changes that occurred in these two treatment groups. Interestingly, differential abundances of S24‐7 bacteria have previously been observed in women with PCOS^15^ and in the LET model,^32^ while changes in the abundance of Rikenellaceae bacteria such as *Alistipes* were previously associated with both LET and DHT treatment.^32^, ^54^ Intriguingly, *M. schaedleri* has not been previously associated with hyperandrogenism or PCOS. *M. schaedleri* is the only known member of the Deferribacteres phylum and is present in low abundance in human and rodent guts. While its role in health and disease is not well understood, it has been paradoxically associated with both protection from *Salmonella*‐induced colitis and vulnerability to colitis in hosts with impaired innate immunity.^59^

In summary, while there has been a strong focus on the role of hyperandrogenism in the development of metabolic dysregulation and insulin resistance in PCOS,^60^ our findings add to a growing body of literature indicating that in certain contexts, elevated androgens are insufficient to induce female metabolic dysfunction. Our results suggest that further investigation into additional factors including changes in aromatase activity that can synergize with hyperandrogenism to drive metabolic dysregulation in females could be informative in understanding PCOS pathology and potential therapeutic interventions. Moreover, our study highlights that treatment with DHT alone or in combination with OVX results in distinct changes in the female gut microbiome. As 16S rRNA gene sequencing analysis provides limited taxonomic resolution, and only indirect functional information, there is a need for future metagenomic, transcriptomic and metabolomic studies to fill this gap. Additionally, as correlative studies cannot distinguish between cause and effect, there is a need for mechanistic studies to determine whether the gut microbiome is required for the development of metabolic dysfunction induced by androgens and if microbial changes induced by hyperandrogenism are sufficient to induce metabolic phenotypes in females in faecal microbiome transplant studies. Moreover, future studies are needed to understand how androgens influence microbial composition and function in females through direct mechanisms in the gut versus indirect mechanisms in host tissues, and how those changes in the gut microbiome influence host physiology.

## AUTHOR CONTRIBUTIONS

**Annie Chen:** Formal analysis (equal); investigation (equal); writing – original draft (equal); writing – review and editing (equal). **Alex Handzel:** Formal analysis (equal); investigation (equal); writing – original draft (equal); writing – review and editing (equal). **Lillian Sau:** Investigation (supporting). **Laura Cui:** Investigation (supporting). **Scott T. Kelley:** Conceptualization (equal); formal analysis (supporting); funding acquisition (supporting); investigation (supporting); supervision (equal); writing – original draft (equal); writing – review and editing (equal). **Varykina G. Thackray:** Conceptualization (equal); formal analysis (supporting); funding acquisition (lead); investigation (supporting); supervision (equal); writing – original draft (equal); writing – review and editing (equal).

## FUNDING INFORMATION

This work was funded by R01 HD095412 to V. G. T. and S. T. K. A. C. was funded by an Endocrine Society Summer Research Fellowship.

## CONFLICT OF INTEREST STATEMENT

The authors declare no conflicts of interest.

## ETHICS STATEMENT

All animal procedures in this study were approved by the University of California, San Diego Institutional Animal Care and Use Committee (Protocol S14011).

## NOVELTY STATEMENT

Studies have shown that hyperandrogenism is correlated with insulin resistance and gut microbial dysbiosis in women with PCOS. They have also shown that hyperandrogenism can result in metabolic dysfunction in rodent models but the mechanisms are not well understood. Our findings add to a growing body of literature indicating that in certain contexts, elevated androgens are insufficient to induce female metabolic dysfunction. Our results suggest that further investigation into additional factors that can synergize with hyperandrogenism to drive metabolic dysregulation in females could be informative in understanding PCOS pathology and potential therapeutic interventions. Moreover, our study highlights that treatment with DHT alone or in combination with OVX results in distinct changes in the gut microbiome, indicating a need for future studies to determine how androgens influence microbial composition and function in females through direct and indirect mechanisms.

## IMPORTANCE OF THE STUDY

As the relationship between hyperandrogenism, insulin resistance and gut dysbiosis may play an important role in PCOS pathophysiology, it is important to understand how androgens contribute to metabolic dysfunction in the context of the pubertal‐to‐adult transition.

## Data Availability

16S rRNA gene sequences are available via the National Center for Biotechnology Information (study accession number PRNA906288). The complete data set, analyses (R Markdown) and results of all the compositional data analysis tests are available on GitHub (https://github.com/Handze1/Differential‐Taxa‐Analysis).

## References

[1] Fauser BC , Tarlatzis BC , Rebar RW , et al. Consensus on women's health aspects of polycystic ovary syndrome (PCOS): the Amsterdam ESHRE/ASRM‐sponsored 3rd PCOS consensus workshop group. Fertil Steril. 2012;97(1):28‐38.e25. 22153789 10.1016/j.fertnstert.2011.09.024

[2] Group TREA‐SPCW . Revised 2003 consensus on diagnostic criteria and long‐term health risks related to polysystic ovary syndrome. Fertil Steril. 2004;81:19‐25. 10.1016/j.fertnstert.2003.10.004 14711538

[3] Boomsma CM , Eijkemans MJ , Hughes EG , Visser GH , Fauser BC , Macklon NS . A meta‐analysis of pregnancy outcomes in women with polycystic ovary syndrome. Hum Reprod Update. 2006;12(6):673‐683. 16891296 10.1093/humupd/dml036

[4] Azziz R , Carmina E , Dewailly D , et al. The androgen excess and PCOS society criteria for the polycystic ovary syndrome: the complete task force report. Fertil Steril. 2009;91(2):456‐488. 18950759 10.1016/j.fertnstert.2008.06.035

[5] Carmina E , Campagna AM , Lobo RA . A 20‐year follow‐up of young women with polycystic ovary syndrome. Obstet Gynecol. 2012;119(2 Pt 1):263‐269. 22270277 10.1097/AOG.0b013e31823f7135

[6] Diamanti‐Kandarakis E , Spritzer PM , Sir‐Petermann T , Motta AB . Insulin resistance and polycystic ovary syndrome through life. Curr Pharm Des. 2012;18(34):5569‐5576. 22834924 10.2174/138161212803307590

[7] Wild RA , Carmina E , Diamanti‐Kandarakis E , et al. Assessment of cardiovascular risk and prevention of cardiovascular disease in women with the polycystic ovary syndrome: a consensus statement by the androgen excess and polycystic ovary syndrome (AE‐PCOS) society. J Clin Endocrinol Metab. 2010;95(5):2038‐2049. 20375205 10.1210/jc.2009-2724

[8] Wild S , Pierpoint T , McKeigue P , Jacobs H . Cardiovascular disease in women with polycystic ovary syndrome at long‐term follow‐up: a retrospective cohort study. Clin Endocrinol. 2000;52(5):595‐600. 10.1046/j.1365-2265.2000.01000.x 10792339

[9] Moran LJ , Misso ML , Wild RA , Norman RJ . Impaired glucose tolerance, type 2 diabetes and metabolic syndrome in polycystic ovary syndrome: a systematic review and meta‐analysis. Hum Reprod Update. 2010;16(4):347‐363. 20159883 10.1093/humupd/dmq001

[10] Churchill SJ , Wang ET , Pisarska MD . Metabolic consequences of polycystic ovary syndrome. Minerva Ginecol. 2015;67(6):545‐555. 26372304

[11] Yang R , Yang S , Li R , Liu P , Qiao J , Zhang Y . Effects of hyperandrogenism on metabolic abnormalities in patients with polycystic ovary syndrome: a meta‐analysis. Reprod Biol Endocrinol. 2016;14(1):67. 27756332 10.1186/s12958-016-0203-8 PMC5069996

[12] Vassilatou E . Nonalcoholic fatty liver disease and polycystic ovary syndrome. World J Gastroenterol. 2014;20(26):8351‐8363. 25024594 10.3748/wjg.v20.i26.8351 PMC4093689

[13] Dunaif A , Segal KR , Futterweit W , Dobrjansky A . Profound peripheral insulin resistance, independent of obesity, in polycystic ovary syndrome. Diabetes. 1989;38(9):1165‐1174. 2670645 10.2337/diab.38.9.1165

[14] Stepto NK , Cassar S , Joham AE , et al. Women with polycystic ovary syndrome have intrinsic insulin resistance on euglycaemic‐hyperinsulaemic clamp. Hum Reprod. 2013;28(3):777‐784. 23315061 10.1093/humrep/des463

[15] Lindheim L , Bashir M , Munzker J , et al. Alterations in gut microbiome composition and barrier function are associated with reproductive and metabolic defects in women with polycystic ovary syndrome (PCOS): a pilot study. PLoS One. 2017;12(1):e0168390. 28045919 10.1371/journal.pone.0168390 PMC5207627

[16] Torres PJ , Siakowska M , Banaszewska B , et al. Gut microbial diversity in women with polycystic ovary syndrome correlates with Hyperandrogenism. J Clin Endocrinol Metab. 2018;103(4):1502‐1511. 29370410 10.1210/jc.2017-02153 PMC6276580

[17] Insenser M , Murri M , Del Campo R , Martinez‐Garcia MA , Fernandez‐Duran E , Escobar‐Morreale HF . Gut microbiota and the polycystic ovary syndrome: influence of sex, sex hormones, and obesity. J Clin Endocrinol Metab. 2018;103(7):2552‐2562. 29897462 10.1210/jc.2017-02799

[18] Qi X , Yun C , Sun L , et al. Gut microbiota–bile acid–interleukin‐22 axis orchestrates polycystic ovary syndrome. Nat Med. 2019;25(8):1225‐1233. 31332392 10.1038/s41591-019-0509-0 PMC7376369

[19] Zeng B , Lai Z , Sun L , et al. Structural and functional profiles of the gut microbial community in polycystic ovary syndrome with insulin resistance (IR‐PCOS): a pilot study. Res Microbiol. 2019;170(1):43‐52. 30292647 10.1016/j.resmic.2018.09.002

[20] Zhang J , Sun Z , Jiang S , et al. Probiotic *Bifidobacterium lactis* V9 regulates the secretion of sex hormones in polycystic ovary syndrome patients through the gut‐brain Axis. mSystems. 2019;4(2):10‐128. 10.1128/mSystems.00017-19 PMC6469956 31020040

[21] Zhou L , Ni Z , Cheng W , et al. Characteristic gut microbiota and predicted metabolic functions in women with PCOS. Endocr Connect. 2020;9(1):63‐73. 31972546 10.1530/EC-19-0522 PMC6993273

[22] Chu W , Han Q , Xu J , et al. Metagenomic analysis identified microbiome alterations and pathological association between intestinal microbiota and polycystic ovary syndrome. Fertil Steril. 2020;113(6):1286‐1298.e4. 32482258 10.1016/j.fertnstert.2020.01.027

[23] Stener‐Victorin E , Padmanabhan V , Walters KA , et al. Animal models to understand the etiology and pathophysiology of polycystic ovary syndrome. Endocr Rev. 2020;41(4):bnaa010. 32310267 10.1210/endrev/bnaa010 PMC7279705

[24] Barontini M , Garcia‐Rudaz MC , Veldhuis JD . Mechanisms of hypothalamic‐pituitary‐gonadal disruption in polycystic ovarian syndrome. Arch Med Res. 2001;32(6):544‐552. 11750729 10.1016/s0188-4409(01)00325-3

[25] Nelson VL , Legro RS , Strauss JF 3rd , McAllister JM . Augmented androgen production is a stable steroidogenic phenotype of propagated theca cells from polycystic ovaries. Mol Endocrinol. 1999;13(6):946‐957. 10379893 10.1210/mend.13.6.0311

[26] Naessen T , Kushnir MM , Chaika A , et al. Steroid profiles in ovarian follicular fluid in women with and without polycystic ovary syndrome, analyzed by liquid chromatography‐tandem mass spectrometry. Fertil Steril. 2010;94(6):2228‐2233. 20171618 10.1016/j.fertnstert.2009.12.081

[27] Xita N , Lazaros L , Georgiou I , Tsatsoulis A . CYP19 gene: a genetic modifier of polycystic ovary syndrome phenotype. Fertil Steril. 2010;94(1):250‐254. 19324338 10.1016/j.fertnstert.2009.01.147

[28] Wang H , Li Q , Wang T , et al. A common polymorphism in the human aromatase gene alters the risk for polycystic ovary syndrome and modifies aromatase activity in vitro. Mol Hum Reprod. 2011;17(6):386‐391. 21282199 10.1093/molehr/gar007

[29] Kauffman AS , Thackray VG , Ryan GE , et al. A novel Letrozole model recapitulates both the reproductive and metabolic phenotypes of polycystic ovary syndrome in female mice. Biol Reprod. 2015;93(3):69. 26203175 10.1095/biolreprod.115.131631 PMC4710190

[30] Esparza LA , Schafer D , Ho BS , Thackray VG , Kauffman AS . Hyperactive LH pulses and elevated kisspeptin and NKB gene expression in the arcuate nucleus of a PCOS mouse model. Endocrinology. 2020;161(4):bqaa018. 32031594 10.1210/endocr/bqaa018 PMC7341557

[31] Skarra DV , Hernandez‐Carretero A , Rivera AJ , Anvar AR , Thackray VG . Hyperandrogenemia induced by Letrozole treatment of pubertal female mice results in hyperinsulinemia prior to weight gain and insulin resistance. Endocrinology. 2017;158(9):2988‐3003. 28911175 10.1210/en.2016-1898 PMC5659661

[32] Kelley ST , Skarra DV , Rivera AJ , Thackray VG . The gut microbiome is altered in a Letrozole‐induced mouse model of polycystic ovary syndrome. PLoS One. 2016;11(1):e0146509. 26731268 10.1371/journal.pone.0146509 PMC4701222

[33] Ho B , Ryback D , Benson B , et al. Gut metabolites are more predictive of disease and cohoused states than gut bacterial features in a polycystic ovary syndrome‐like mouse model. mSystems. 2021;6(5):e0114920. 34519532 10.1128/mSystems.01149-20 PMC8547464

[34] Torres PJ , Ho BS , Arroyo P , et al. Exposure to a healthy gut microbiome protects against reproductive and metabolic dysregulation in a PCOS mouse model. Endocrinology. 2019;160:1193‐1204. 30924862 10.1210/en.2019-00050 PMC6482036

[35] Walters KA . Androgens in polycystic ovary syndrome: lessons from experimental models. Curr Opin Endocrinol Diabetes Obes. 2016;23(3):257‐263. 26866639 10.1097/MED.0000000000000245

[36] Padmanabhan V , Veiga‐Lopez A . Animal models of the polycystic ovary syndrome phenotype. Steroids. 2013;78(8):734‐740. 23701728 10.1016/j.steroids.2013.05.004 PMC3700672

[37] Caldwell AS , Middleton LJ , Jimenez M , et al. Characterization of reproductive, metabolic, and endocrine features of polycystic ovary syndrome in female hyperandrogenic mouse models. Endocrinology. 2014;155(8):3146‐3159. 24877633 10.1210/en.2014-1196

[38] van Houten EL , Kramer P , McLuskey A , Karels B , Themmen AP , Visser JA . Reproductive and metabolic phenotype of a mouse model of PCOS. Endocrinology. 2012;153(6):2861‐2869. 22334715 10.1210/en.2011-1754

[39] Marcondes FK , Bianchi FJ , Tanno AP . Determination of the estrous cycle phases of rats: some helpful considerations. Braz J Biol. 2002;62(4A):609‐614. 12659010 10.1590/s1519-69842002000400008

[40] Caporaso JG , Lauber CL , Walters WA , et al. Ultra‐high‐throughput microbial community analysis on the Illumina HiSeq and MiSeq platforms. ISME J. 2012;6(8):1621‐1624. 22402401 10.1038/ismej.2012.8 PMC3400413

[41] Callahan BJ , McMurdie PJ , Rosen MJ , Han AW , Johnson AJ , Holmes SP . DADA2: high‐resolution sample inference from Illumina amplicon data. Nat Methods. 2016;13(7):581‐583. 27214047 10.1038/nmeth.3869 PMC4927377

[42] Gloor GB , Macklaim JM , Pawlowsky‐Glahn V , Egozcue JJ . Microbiome datasets are compositional: and this is not optional. Front Microbiol. 2017;8:2224. 29187837 10.3389/fmicb.2017.02224 PMC5695134

[43] Sisk‐Hackworth L , Kelley ST . An application of compositional data analysis to multiomic time‐series data. NAR Genom Bioinform. 2020;2(4):lqaa079. 33575625 10.1093/nargab/lqaa079 PMC7671389

[44] Breiman L . Random forests. Mach Learn. 2001;45(1):5‐32.

[45] Esparza LA , Terasaka T , Lawson MA , Kauffman AS . Androgen suppresses in vivo and in vitro LH pulse secretion and neural Kiss1 and Tac2 gene expression in female mice. Endocrinology. 2020;161(12):bqaa191. 33075809 10.1210/endocr/bqaa191 PMC7671291

[46] Andrisse S , Childress S , Ma YP , et al. Low‐dose Dihydrotestosterone drives metabolic dysfunction via cytosolic and nuclear hepatic androgen receptor mechanisms. Endocrinology. 2017;158(3):531‐544. 27967242 10.1210/en.2016-1553 PMC5460775

[47] Xue P , Wang Z , Fu X , et al. A Hyperandrogenic mouse model to study polycystic ovary syndrome. J Vis Exp. 2018;140:e58379. 10.3791/58379 PMC6235414 30346398

[48] Caldwell ASL , Edwards MC , Desai R , et al. Neuroendocrine androgen action is a key extraovarian mediator in the development of polycystic ovary syndrome. Proc Natl Acad Sci U S A. 2017;114(16):E3334‐E3343. 28320971 10.1073/pnas.1616467114 PMC5402450

[49] Rodriguez Paris V , Edwards MC , Aflatounian A , et al. Pathogenesis of reproductive and metabolic PCOS traits in a mouse model. J Endocr Soc. 2021;5(6):bvab060. 34056500 10.1210/jendso/bvab060 PMC8152184

[50] Riant E , Waget A , Cogo H , Arnal JF , Burcelin R , Gourdy P . Estrogens protect against high‐fat diet‐induced insulin resistance and glucose intolerance in mice. Endocrinology. 2009;150(5):2109‐2117. 19164473 10.1210/en.2008-0971

[51] Roland AV , Nunemaker CS , Keller SR , Moenter SM . Prenatal androgen exposure programs metabolic dysfunction in female mice. J Endocrinol. 2010;207(2):213‐223. 20713501 10.1677/JOE-10-0217 PMC3612271

[52] Manti M , Fornes R , Pironti G , et al. Maternal androgen excess induces cardiac hypertrophy and left ventricular dysfunction in female mice offspring. Cardiovasc Res. 2020;116(3):619‐632. 31382275 10.1093/cvr/cvz180

[53] Torres PJ , Skarra DV , Ho BS , et al. Letrozole treatment of adult female mice results in a similar reproductive phenotype but distinct changes in metabolism and the gut microbiome compared to pubertal mice. BMC Microbiol. 2019;19(1):57. 30871463 10.1186/s12866-019-1425-7 PMC6419356

[54] Zheng Y , Yu J , Liang C , Li S , Wen X , Li Y . Characterization on gut microbiome of PCOS rats and its further design by shifts in high‐fat diet and dihydrotestosterone induction in PCOS rats. Bioprocess Biosyst Eng. 2021;44(5):953‐964. 32157446 10.1007/s00449-020-02320-w

[55] Zhang F , Ma T , Cui P , et al. Diversity of the gut microbiota in Dihydrotestosterone‐induced PCOS rats and the pharmacologic effects of Diane‐35, probiotics, and Berberine. Front Microbiol. 2019;10:175. 30800111 10.3389/fmicb.2019.00175 PMC6375883

[56] Rodriguez Paris V , Wong XYD , Solon‐Biet SM , et al. The interplay between PCOS pathology and diet on gut microbiota in a mouse model. Gut Microbes. 2022;14(1):2085961. 35787106 10.1080/19490976.2022.2085961 PMC9450977

[57] Choi S , Hwang YJ , Shin MJ , Yi H . Difference in the gut microbiome between Ovariectomy‐induced obesity and diet‐induced obesity. J Microbiol Biotechnol. 2017;27(12):2228‐2236. 29121700 10.4014/jmb.1710.10001

[58] Wang F , Yu P , Gui X , Wang Y , Xue C , Wang J . Sialoglycoprotein isolated from the eggs of *Carassius auratus* prevents bone loss: an effect associated with the regulation of gut microbiota in ovariectomized rats. Food Funct. 2016;7(12):4764‐4771. 27808338 10.1039/c6fo01103a

[59] Herp S , Durai Raj AC , Salvado Silva M , Woelfel S , Stecher B . The human symbiont *Mucispirillum schaedleri*: causality in health and disease. Med Microbiol Immunol. 2021;210(4):173‐179. 34021796 10.1007/s00430-021-00702-9 PMC7615636

[60] Sanchez‐Garrido MA , Tena‐Sempere M . Metabolic dysfunction in polycystic ovary syndrome: pathogenic role of androgen excess and potential therapeutic strategies. Mol Metab. 2020;35:100937. 32244180 10.1016/j.molmet.2020.01.001 PMC7115104

